# DNA-PKcs orchestrates CTLA-4 depletion-induced senescence in cancer cells

**DOI:** 10.1038/s41419-026-08419-4

**Published:** 2026-02-04

**Authors:** Je-Jung Lee, Woo Joong Rhee, So Young Kim, Jisun Lee, Su Ful Jung, Jooyeon Oh, In Ho Park, Jeon-Soo Shin

**Affiliations:** 1 Department of Microbiology, Yonsei University College of Medicine, Seoul, South Korea; 2 Institute for Immunology and Immunological Diseases, Yonsei University College of Medicine, Seoul, South Korea; 3 Brain Korea 21 FOUR Project for Medical Science, Yonsei University College of Medicine, Seoul, South Korea; 4 Department of Biomedical Sciences, Yonsei University College of Medicine, Seoul, South Korea

**Keywords:** Senescence, Experimental models of disease

## Abstract

Immune checkpoints such as cytotoxic T-lymphocyte–associated protein 4 (CTLA-4), programmed cell death 1 (PD-1), and programmed cell death ligand 1 (PD-L1) have been targeted in cancer therapy, however, the efficacy of these interventions remains limited. Beyond its immune function on T cell surfaces, CTLA-4 is also expressed in various intrinsic cancer cells, where it influences cell proliferation, metastasis, and apoptosis. The present study aimed to investigate the function of CTLA-4 in cancer cells by investigating the consequences of CTLA-4 depletion in melanoma cells. We found that targeting CTLA-4 in melanoma cells inhibited proliferation via the induction of senescence, which was attributed to genomic instability resulting from a decrease in Aurora B expression, leading to the activation of the DNA-dependent protein kinase catalytic subunit (DNA-PKcs)–stimulator of interferon genes (STING) pathway. Notably, DNA-PKcs coordinates CTLA-4 depletion–induced senescence by regulating the STING pathway. Mouse study showed that the tumor suppressive effect of CTLA-4 depletion in allograft cancer models via senescence induction. Furthermore, public data analysis showed a negative correlation between CTLA-4 and DNA-PKcs expressions in patients. Conclusively, CTLA-4-depletion induces senescence via genome instability, which activates DNA-PKcs and ultimately leads to cancer growth regression. These findings suggest that intracellular CTLA-4 targeting can confer to cancer therapy.

**Figure:**
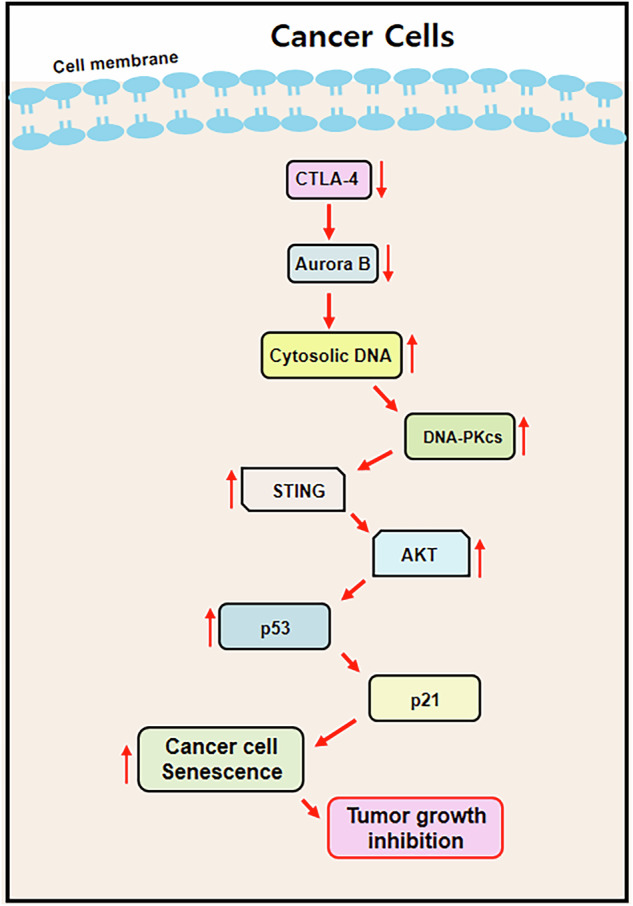
CTLA-4 depletion-induced senescence in cancer. CTLA-4 depletion-induced senescence in cancer. CTLA-4 deficiency induces senescence via the DNA PKcs-STING-AKT pathway in cancer cells. When CTLA-4 is depleted in cancer cells, the genome becomes unstable due to the reduction of Aurora B expression, then consequently DNA damage occurs accompanied by micronuclei formation in the cytosol. Subsequently, DNA-PKcs is activated and sequentially promotes the STING-AKT-p21 signaling pathway, which mediates cellular senescence and eventually prevents tumor growth.

CTLA-4 depletion-induced senescence in cancer. CTLA-4 depletion-induced senescence in cancer. CTLA-4 deficiency induces senescence via the DNA PKcs-STING-AKT pathway in cancer cells. When CTLA-4 is depleted in cancer cells, the genome becomes unstable due to the reduction of Aurora B expression, then consequently DNA damage occurs accompanied by micronuclei formation in the cytosol. Subsequently, DNA-PKcs is activated and sequentially promotes the STING-AKT-p21 signaling pathway, which mediates cellular senescence and eventually prevents tumor growth.

## Introduction

Cellular senescence is a state of permanent cell cycle arrest characterized by morphological changes, increased activity of senescence-associated β-galactosidase (SA-β-Gal) at pH 6.0, and the induction of the expression of cell cycle checkpoints like p21, p16, and p27 [1]. The expression of heterochromatin and DNA damage markers, including H3K9 trimethylation (H3K9me3) and γ-H2AX [2], is also linked to senescence. Cellular senescence has been demonstrated to impede tumor growth by arresting the cell cycle [3].

While cytotoxic T-lymphocyte–associated protein 4 (CTLA-4), a key regulator of immune responses, is expressed on the T cell surface and acts as a negative regulator of T cell proliferation, it is also expressed in various cancers [4] including numerous human and mouse melanoma cell lines, primary melanomas, melanoma stem cells, and normal melanocytes [4–6]. Its expression is associated with poor prognosis. Consequently, evidence of tumor-related CTLA-4 functions has prompted investigations into the cell-intrinsic effects of CTLA-4 in tumors. CTLA-4 primarily accumulates in intracellular compartments and transiently relocates to the cell surface upon stimulation before being rapidly internalized [7]. Despite this, the cytoplasmic function of CTLA-4 remains poorly understood, and its role, particularly in cancer cells, needs to be re-evaluated.

The cyclic GMP–AMP synthase (cGAS)-stimulator of interferon genes (STING) is an important mediator of inflammation, cellular stress, tissue damage, and senescence [8, 9]. The process of cGAS detection and binding to DNA is initiated by the presence of DNA in the cytosol, which may be due to infection, separation from self DNA damage, or slippage during replication. This process subsequently engages the adaptor protein STING and activates TANK-binding kinase 1 (TBK1). TBK1 has shown to phosphorylate STING and the interferon regulatory factor 3 (IRF3), leading to the dimerization and subsequent nuclear translocation of IRF3, which in turn induces the production of type I interferons [10]. The cGAS-STING pathway has also been implicated in the process of senescence. The occurrence of genomic or epigenomic changes due to genotoxic stress results in the formation of cytoplasmic chromatin fragments (CCFs) by senescent cells, thereby triggering the cGAS-STING pathway [9, 11]. Consequently, STING signaling emerges as a pivotal pathway in senescence, representing a potential therapeutic strategy for suppressing tumors through the induction of senescence.

Serine/threonine kinase AKT has been shown to enhance the expression of p53 and p21, increase cell size, and induce senescence [12, 13]. While the PI3K-AKT-mTORC1 pathway is essential for cell proliferation, persistent hyperactivation of this pathway leads to cellular senescence, serving as a tumor-suppressive mechanism that prevents transformation. Furthermore, the interaction between the AKT and STING pathways [14, 15] has expanded the previous limited notion of AKT to the STING-related fields of senescence [14, 16]. The DNA-dependent protein kinase catalytic subunit (DNA-PKcs) serves as a pivotal sensor for DNA double-strand breaks (DSBs) and functions in non-homologous end joining (NHEJ) DNA damage repair (DDR) pathways alongside Ku70/Ku80. Beyond its involvement in NHEJ, DNA-PKcs also contributes to STING-dependent and STING-independent pathways for intracellular nucleic acid recognition. While DNA-PKcs is predominantly nuclear, its non-nuclear functions are critical for maintaining genomic integrity and DNA fidelity [17]. The kinase activity of DNA-PKcs is vital in various diseases, including cancers, because of its involvement in cellular processes such as cell death, cell division, and senescence [18]. In addition to its well-established role in DNA damage response, the association of DNA-PKs with the STING-related pathway has significant implications for cancer therapy [19, 20].

In the present study, we aimed to investigate the role of CTLA-4 in cancer cell senescence by intrinsic CTLA4-depletion via a signaling cascade involving DNA damage by genomic instability, ultimately leading to tumor regression in melanoma cells.

## Materials and methods

### Cell culture, transfection, and reagents

A375 human melanoma, B16-F10 mouse melanoma, and BJ human foreskin cells were cultured in Dulbecco’s Modified Eagle Medium (DMEM) supplemented with 10% fetal bovine serum and 1% penicillin-streptomycin. The CTLA-4 and Aurora B plasmids were obtained from Sino Biological. Plasmids and short interfering (si)RNA transfections were performed using FuGene HD reagent and RNAiMAX, respectively, as recommended by the manufacturers. siRNA duplexes against human CTLA-4, DNA-PKcs, human and mouse STING, AKT, and control siRNA were purchased from Bioneer Inc. siRNA duplexes against mouse CTLA-4 and DNA-PK were obtained from Santa Cruz Biotechnology. Doxorubicin (Dox) and cisplatin were obtained from Calbiochem and Sigma-Aldrich, respectively. Nu7441 and KU-60019 were purchased from Selleck Chemicals.

### Western blotting (WB) analysis

WB analysis was followed as previously described [21]. The following antibodies were used in this study: p-IRF3, STING, p-TBK1, p-AKT, AKT, p-mTOR, mTOR, β-actin, and H3K9me3 from Cell Signaling Technologies, Inc.; CTLA-4, DNA-PKcs, γ-H2AX, and p16 from Abcam; p21 and p27 from BD Biosciences; p53 from Santa Cruz Biotechnology, p-STING from Affinity; and antibody to BRAF^V600E^ from Sigma-Aldrich. Secondary antibodies for immunofluorescence were also obtained from Cell Signaling Technologies. Antibody-antigen complexes were detected using HRP-conjugated secondary antibodies and visualized using a standard chemiluminescence method according to the manufacturer’s instructions.

### Cell cycle analysis

Cells were collected by trypsinization, fixed in 70% ethanol, washed in phosphate-buffered saline (PBS), and resuspended in 1 ml of PBS containing 1 µg/ml RNase and 50 µg/ml propidium iodide (PI). After incubation in the dark for 30 min, cell cycle distributions were analyzed using BD FACS Verse II (Becton Dickinson, Franklin Lakes Enterprises, NJ, US). The data were assessed using FLOWJO, Single Cell Analysis Software V10 (OR, USA).

### Cell morphology analysis and SA-β-Gal staining

Cellular morphologies were analyzed by captured using an inverted phase-contrast microscope (Olympus, Tokyo, Japan). SA-β-Gal staining was performed as recommended by the manufacturers (CELLEvent^TM^ Senescence Green Detection Kit, C10850, Invitrogen, Waltham, MA, USA) or as previously described [22]. The examinations were performed on day 3 following each treatment, unless otherwise indicated.

### Confocal microscopy

Confocal microscopy assay was followed as previously described **[**21] using antibodies against CTLA-4, p-AKT, p-mTOR, DNA-PKcs, p21, and γ-H2AX (same as those used for WB).

### Chromatin immunoprecipitation (ChIP) analysis

ChIP assay was followed as previously described **[**21, 23].

### Generation of B16-F10^CTLA-4 KO^ cell line

CRISPR/Cas9-mediated knockout of CTLA-4 in the B16-F10 cell line was generated by using CTLA-4 Double Nickase Plasmid (m) (sc-419533-NIC; Santa Cruz Biotechnology) following the manufacturer’s instructions. In detail, the CRISPR/Cas9 plasmid was transfected to the B16-F10^WT^ cells, and the transfected cells were selected using a normal growth medium containing puromycin (Santa Cruz Biotechnology). After the antibiotic selection was completed, the cells expressing GFP fluorescence were sorted using BD FACS Aria II sorter (Becton Dickinson) and seeded into the 96-well plates, with one cell per well. After selecting and verifying successful gene deletion among clones, the CTLA-4 deficient (KO) B16-F10^CTLA-4 KO^ cells were utilized for subsequent experiments.

### Mouse experiment

All animal procedures were approved by the Institutional Animal Care and Use Committee (IRB No. 2023-0132). Briefly, seven-to-eight-week-old female C57BL/6 mice were housed in a specific pathogen-free facility and used for allograft tumor experiments. To generate tumors, 1 × 10^6^ B16-F10^WT^ and B16-F10^CTLA-4 KO^ cells were suspended in 100 μL of PBS, respectively, were injected into both sides of the dorsal subcutaneous area of the mice, and tumor masses were successfully formed after implantation. For Dox treatment, mice were administered a single intraperitoneal injection of Dox (9 mg/kg body weight) after tumor formation, and the tumors were collected after seven days. The tumor tissues were fixed overnight in 4% formalin, embedded in paraffin, and subjected to immunohistochemistry to detect the expression of STING, H3K9me3, and p-IRF3. The sections were counterstained with DAPI. Photographs were acquired in randomly chosen fields per tumor section according to standard procedures.

### Analysis of publicly available data

The mRNA expression z-scores of The Cancer Genome Atlas (TCGA) Pan-Cancer Atlas were obtained from the cBioPortal for Cancer Genomics (https://www.cbioportal.org). The data of patients with skin cutaneous melanoma, lung squamous cell carcinoma, cervical squamous cell carcinoma, and head and neck squamous cell carcinoma were used for analysis. To analyze the correlation between different genes, Pearson’s correlation test was used. Additionally, based on the CTLA-4 expression level, patients were categorized into the CTLA-4-high and CTLA-4-low groups, and the gene expression levels were compared between the groups.

### Statistical analysis

Statistical analysis was performed as previously described [21]. Data are represented as the mean ± standard deviation (SD). Asterisks denote the *p*-values as follows: *p < 0.05, **p < 0.01, and ***p < 0.001.

## Results

### CTLA-4 depletion induces senescence in melanoma cells

**Fig. 1:**
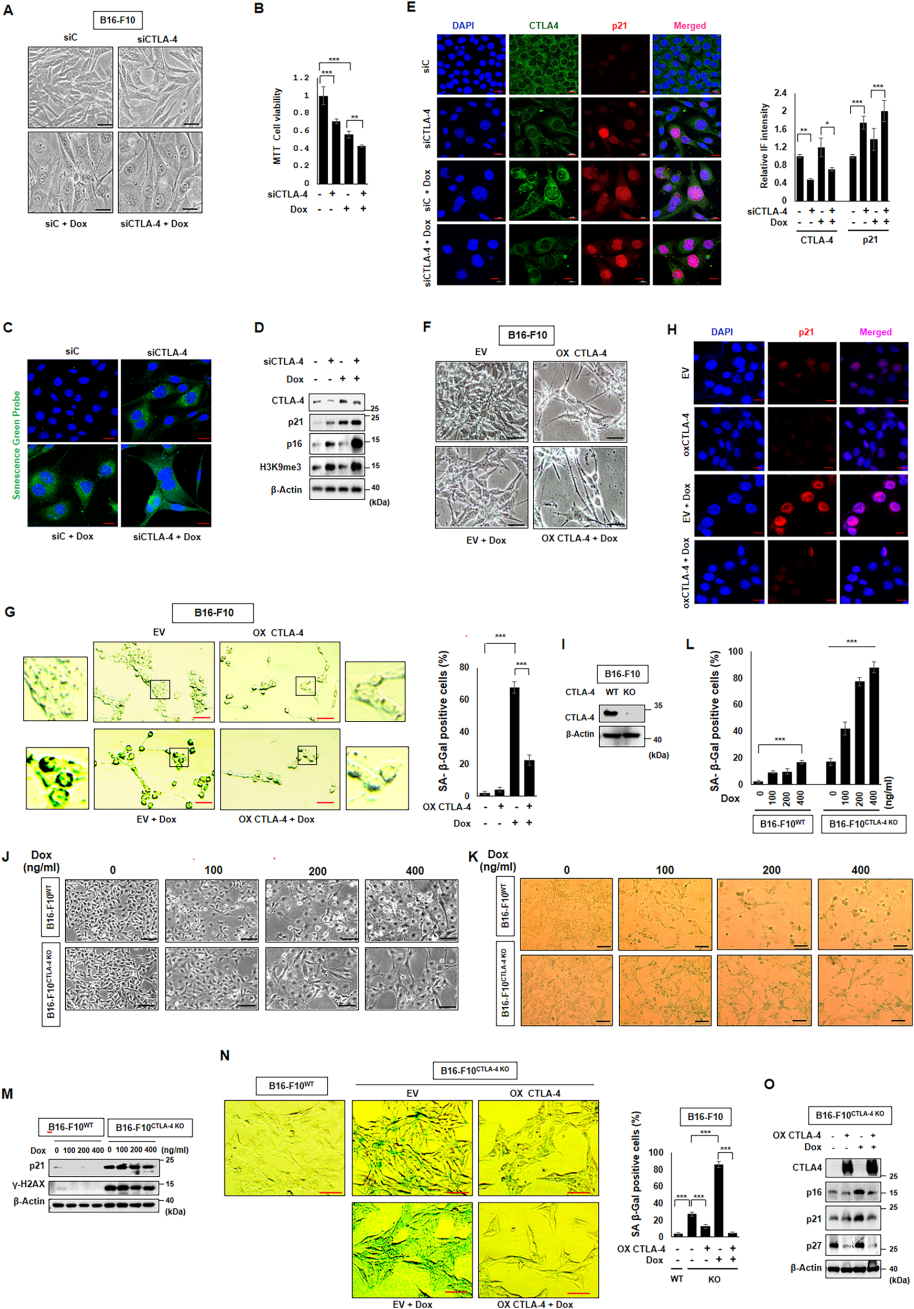
CTLA-4-depletion induces senescence in mouse melanoma cells. B16-F10 cells were transfected with 100 nM siC and CTLA-4 siRNA, respectively, one day before treatment with or without 100 ng/ml Dox. Subsequently, morphological changes were detected (**A**) cell viability by MTT assay (**B**) SA-β-Gal staining detected by Senescent Green Probe (**C**) WB with indicated antibodies (**D**) confocal microscopy assay for CTLA-4, p21, and DAPI levels followed by quantification (**E**) were performed on day two after Dox treatment. CTLA-4 was overexpressed in B16-F10 one day before treatment with or without Dox (**F**–**H**). Then, morphological change (**F**) SA-β-Gal staining with quantification (**G**) and confocal microscopy assay for p21 and DAPI (**H**) were performed on day 2 post Dox treatment. B16-F10^CTLA-4 KO^ cells were generated and confirmed with anti-CTLA-4 antibody (**I**). B16-F10^wt^ and B16-F10^CTLA-4 KO^ cells were treated with different Dox concentration such as 0, 100, 200, 400 ng/ml, and morphological changes (**J**), SA-β-Gal staining (**K**) with quantification (**L**) and WB with indicated antibodies (**M**) were performed on day 2 post Dox treatment. CTLA-4 was reconstituted in B16-F10^CTLA-4 KO^ cells, and SA-β-Gal staining with quantification (**N**), and WB with indicated antibodies (**O**) were performed on day two post Dox treatment. Scale bars, 50 μm (**A**, **F**, **G**, **J**, **K**) 20 μm (**C**, **E**, **H**). The significance of the statistical differences among the four groups was calculated using a one-way analysis of variance and Newman–Keuls methods. Quantitative data are expressed as means ± SD. *N* = 3, *p < 0.05. **p < 0.01. ***p < 0.001.

To investigate the role of CTLA-4 in cancer cell senescence, we silenced CTLA-4 alone or together with a low dose doxorubicin (Dox), an anti-cancer drug used to induce senescence [24, 25], in B16-F10 mouse melanoma cells, which express high levels of CTLA-4 [4, 26]. Unexpectedly, CTLA-4 silencing induced senescence phenotypes, including increased cell size, decreased cell viability as assessed by the MTT assay, and elevated SA-β-Gal activity as indicated by a senescent green probe (Fig. 1A–C). Additionally, senescence markers such as the cell cycle checkpoints p21 and p16 and the heterochromatin marker H3K9me3 expressions were elevated compared to controls as shown by western blotting (Fig. 1D). Confocal microscopy analysis also demonstrated an inverse correlation between CTLA-4 and p21 expressions (Fig. 1E). All those effects by CTLA-4 siRNA treatment were promoted by Dox co-treatment (Fig. 1A–E). Next, we conducted experiments where CTLA-4 was overexpressed in B16-F10 cells. Intriguingly, even sole CTLA-4 overexpression induced apoptotic-like morphology, and the senescence morphology induced by Dox was disrupted (Fig. 1F), which is accompanied by the decrease of SA-β-Gal activity and p21 expression, where the increased SA-β-Gal activity and p21 expression by Dox treatment were also aborted by CTLA-4 overexpression (Fig. 1G, H). In addition, CTLA-4 knockout (KO) B16-F10 (B16-F10^CTLA-4 KO^) cells, which were generated by CRISPR/Cas9 system, showed senescence-like morphology and SA-β-Gal activity, whereas WT B16-F10 (B16-F10^WT^) showed apoptotic-like morphology to Dox in a dose-dependent manner (Fig. 1I–L). And senescence markers, p21 and γ-H2AX expressions were much higher in B16-F10^CTLA-4 KO^ cells (Fig. 1M). Additionally, CTLA-4 complementation in B16-F10^CTLA-4 KO^ cells interrupted SA-β-Gal positivity induced by Dox (Fig. 1N). And cell cycle inhibitors, p16, p21, and p27, were aborted when CTLA-4 was overexpressed (Fig. 1O).

We employed human melanoma cell line A375 to verify these results. Similar to B16-F10 mouse cells, A375 cells showed the increased cell size, a decrease in cell viability, an increase of SA-β-Gal activity, and the increased levels of p21 and p16 when CTLA-4 siRNA was treated (Fig. S1A–E). All of those effects by CTLA-4 siRNA were promoted by Dox co-treatment (Fig. S1A–E). When CTLA-4 was overexpressed in A375 cells, SA-β-Gal activity and p21 expression led by Dox were decreased (Fig. S1F, G). To observe the effect of CTLA-4 knockdown on apoptosis, we performed an Annexin V/PI staining analysis along with a positive control using cisplatin (CPT). As shown in Fig. S1H, I, and no significant apoptosis was observed. Next, we investigated the effects of depleting CTLA-4 in non-tumoral cells using human skin fibroblast BJ cells. BJ cells showed low CTLA-4 expression, and after CTLA-4 siRNA transfection, they exhibited senescence-like morphological changes and p21 upregulation (Fig. S1J, K). Furthermore, the most frequent melanoma tumors are B-Raf mutated. Given this, we investigated the effects of depleting CTLA-4 in B-Raf mutated melanoma cells. A375 cells are known to carry BRAF^V600E^, a common mutant form of BRAF [27]. Interestingly, BRAF^V600E^ mutant expression levels were elevated along with p21 levels in A375 CTLA-4 KO melanoma cells (Fig. S1L). This is consistent with previous reports that BRAF^V600E^ mutant induces senescence [28, 29].

All together, targeting intrinsic CTLA-4 in melanoma A375 and non-tumoral BJ cells induces cellular senescence and halts the proliferation of cancer cells.

### Aurora B reduction by CTLA-4 depletion confers senescence induction via cell cycle arrest through genome instability marked with micronuclei

**Fig. 2:**
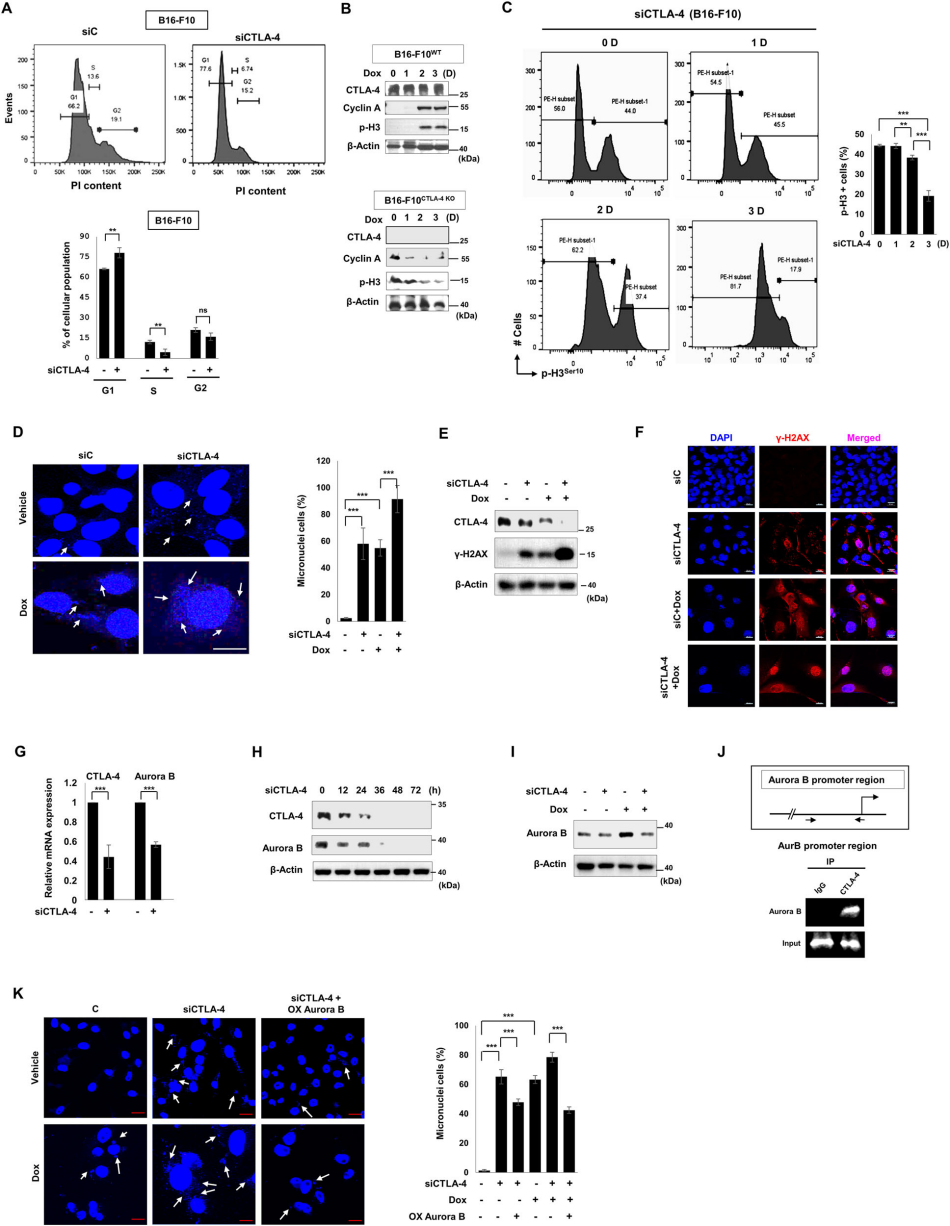
AURKB reduction by CTLA-4 depletion contributes to senescence induction via cell cycle arrest through genome instability in cancer cells. B16-F10 cells were treated with CTLA-4 siRNA, and cell cycle analysis with quantification (**A**). B16-F10^wt^ and B16-F10^CTLA-4 KO^ cells were treated with 100 ng/ml Dox as indicated times, and WB was performed with indicated antibodies (**B**). Cells were transfected with CTLA-4 siRNA, then FACS analysis for p-H3 was performed and quantified from day one to three post siRNA treatment (**C**). DAPI staining for micronuclei assay with quantification (**D**). WB with indicated antibodies (**E**) and confocal microscopy assay for γ-H2AX (**F**) were performed on day two post Dox treatment. Cells were treated with CTLA-4 siRNA, and real-time PCR for CTLA-4 and Aurora B was performed (**G**) and WB (**H**) was performed as indicated times post CTLA-4 siRNA transfection. CTLA-4 siRNA transfection was treated one day before Dox treatment, and WB was performed as indicated antibodies (**I**). Chromatin immunoprecipitation assay (ChIP) was performed with an anti-IgG or CTLA-4 antibody, respectively. PCR was performed using the indicated Aurora B promoter-specific primers for ChIP assays in A375 cells (**J**). Aurora B was overexpressed one day before CTLA-4 siRNA treatment in B16-F10 cells, and DAPI staining for micronuclei assay and quantification (**K**) was performed on day 3 post CTLA-4 siRNA treatment. Scale bars, 20 μm (**D**, **F**, **K**). The significance of the statistical differences among the four groups was calculated using a one-way analysis of variance and Newman–Keuls methods. Quantitative data are expressed as means ± SD. *N* = 3, *p < 0.05. **p < 0.01. ***p < 0.001.

The expression of CTLA-4 influenced the cell proliferation, therefore, we checked cell cycle status. When B16-F10 cells were transfected with CTLA-4 siRNA, G1 phase was increased with the decrease of S phase. (Fig. 2A). While cyclin A and phospho-histone 3 (p-H3) as proliferation markers were increased in B16-F10^WT^ cells, both were decreased in B16-F10^CTLA-4 KO^ cells after Dox treatment (Fig. 2B). And p-H3 expressions after CTLA-4 silencing was declined in a time-dependent manner in FACS analysis (Fig. 2C). The micronuclei formation and DNA damage marker γ-H2AX expression were increased upon CTLA-4 silencing, particularly combined with Dox treatment (Fig. 2D–F), implying that micronuclei can be derived as a result of DNA damage caused by genomic instability due to CTLA-4 depletion.

Aurora B has been known for its role in maintaining genome stability and preventing micronuclei formation [25, 26], and phosphorylating H3 during mitosis [30]. Inhibition of Aurora B can cause cell cycle arrest and genome instability [31, 32]. Therefore, we doubted the Aurora B status in CTLA-4 depleted cells. Indeed, Aurora B was downregulated at the mRNA and protein level upon CTLA-4 siRNA treatment in our experiments (Fig. 2G–I). Additionally, CTLA-4 bound to Aurora B promoter region (Fig. 2J) was proven by chromatin immunoprecipitation (ChIP) assay, which indicates that Aurora B reduction by CTLA-4 depletion is due to the transcription regulation. Furthermore, micronuclei induced by CTLA-4 siRNA alone or in combination with Dox were attenuated by Aurora B overexpression in B16-F10 cells (Fig. 2K).

We confirmed above results in A375 human melanoma cells. The cell cycle analysis, the change of cyclin A and p-H3, micronuclei formation, and γ-H2AX expression were similar to B16-F10 when CTLA-4 was silenced (Fig. S2A–D). Furthermore, Aurora B mRNA or protein expression was downregulated upon CTLA-4 siRNA treatment and elevated by CTLA-4 overexpression (Fig. S2E–G). All these findings collectively suggest that CTLA-4 depletion leads to a decrease in Aurora B levels, resulting in micronuclei formation and DNA damage.

### CTLA-4-depletion activates DNA-PKcs that governs STING signaling

**Fig. 3:**
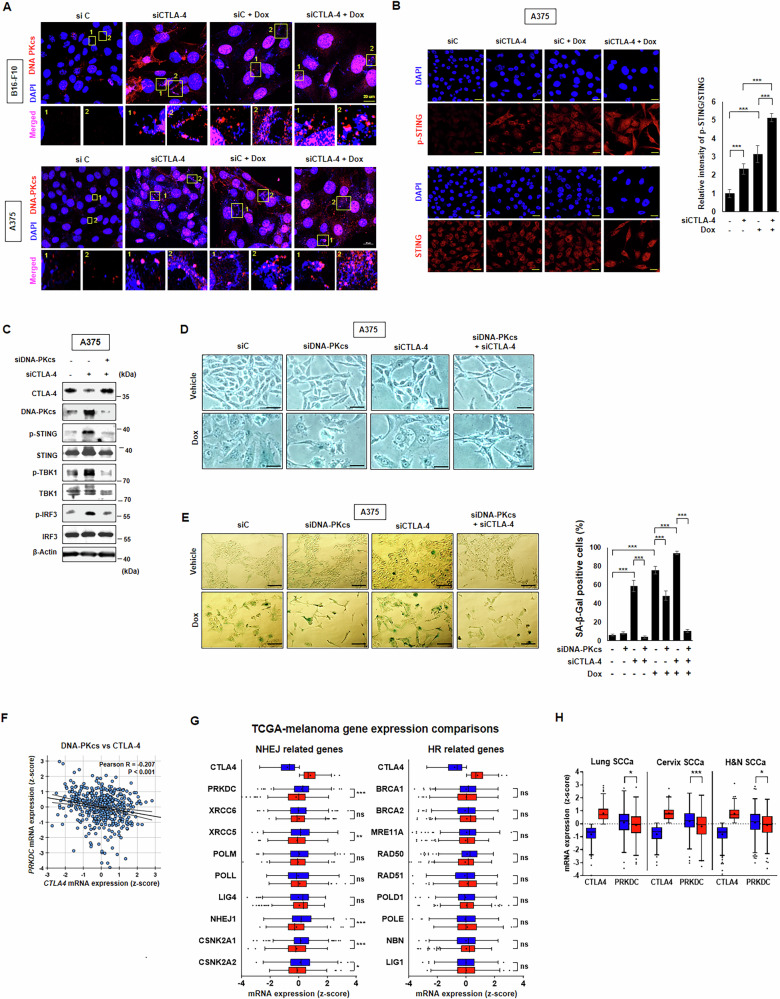
DNA-PKcs governs STING signaling in DNA damage responses induced by CTLA-4-depletion. B16-F10 and A375 cells were treated with 100 nM siC and CTLA-4 siRNA, respectively, one day before treatment with or without 100 ng/ml Dox. Then, confocal microscopy assays for DNA-PKcs and DAPI (**A**) and p-STING with STING (**B**) were performed on day two post Dox treatment. The relative intensity of p-STING/STING from (**B**) was quantified. A375 cells were treated with 100 nM siC and siDNA-PKcs, respectively, one day before treatment with or without 100 nM CTLA-4 siRNA. On day three post CTLA-4 siRNA treatment, WB with indicated antibodies (**C**) morphological changes (**D**) and SA-β-Gal staining with quantification by counting positive cells among 100 cells (*n* = 3) (**E**). Public data analysis involved examining the correlation of CTLA-4 with DNA-PKcs. The mRNA expression z-scores from The Cancer Genome Atlas (TCGA) PanCancer Atlas were obtained from the cBioPortal for Cancer Genomics. The correlation between the mRNA expression of PRKDC and CTLA4 (**F**). Patients were categorized into high (top 1/2, red color) and low (bottom 1/2, blue color) CTLA-4 expression groups. The correlation with non-homologous end joining (NHEJ) pathway components, including DNA-PKcs, and HR components in skin cutaneous melanoma patients was analyzed (**G**). The analysis also included data from patients with lung squamous cell carcinoma (Lung SCCa), cervical squamous cell carcinoma (Cervix SCCa), and head and neck squamous cell carcinoma (H&N SCCa) (**H**). Scale bars: 20 μm (**A**, **B**), 50 μm (**D**, **E**). The significance of the statistical differences among four or eight groups was calculated using one-way analysis of variance and the Newman–Keuls test. Quantitative data are expressed as means ± SD. Asterisks denote the *p*-values: *p < 0.05, **p < 0.01, ***p < 0.001. For public data, statistical analysis was performed using GraphPad Prism 5 (GraphPad Software, Inc., La Jolla, CA, USA) and SPSS Statistics 26.0 (IBM, Armonk, NY, USA). Two-tailed, unpaired Student’s t-tests were used to determine the statistical significance between the two groups. The significance of the statistical differences among three or more groups was calculated using a one-way analysis of variance and the Newman–Keuls test. The correlation between different genes was analyzed using Pearson’s correlation test. Data are shown as the mean ± standard deviation (SD). Asterisks denote *p*-values: *p < 0.05, **p < 0.01, ***p < 0.001.

DNA-PKcs is required for cGAS/STING-dependent or -independent DNA sensing pathway [19, 33]. We first investigated whether DNA-PKcs is involved in senescence induced by CTLA-4 depletion. DNA-PKcs was found to be co-localized with micronuclei in B16-F10 and A375 cells and was upregulated by CTLA-4 silencing, which was further enhanced by Dox co-treatment in confocal microscopy assays (Fig. 3A). Concurrently, the expression of p-STING, which is increased in senescence, was increased by CTLA-4 siRNA transfection whereas STING showed similar level in A375 cells using confocal analysis (Fig. 3B). And the elevated p-STING and its downstream molecules of p-TBK-1 and p-IRF3 by CTLA-4 depletion were decreased to baseline level by siDNA-PKcs transfection (Fig. 3C). The senescence-like morphology and SA-β-Gal positivity induced by CTLA-4 depletion was significantly attenuated upon silencing of DNA-PKcs (Fig. 3D, E). These results suggest that CTLA-4 deficiency leads to STING activation via DNA-PKcs employing to induce senescence. Next, we analyzed TCGA data from patients with skin cutaneous melanoma, and found a negative correlation between the mRNA expression of PRKDC, which encodes DNA-PKcs, and CTLA4 (Fig. 3F). Moreover, patients were stratified into high- and low-CTLA-4 expression groups based on the median CTLA-4 levels, revealing an inverse correlation with components of the NHEJ pathway, which is related to DNA-PKcs, but not with the homologous recombination (HR) pathway (Fig. 3G). These findings were consistent across TCGA datasets from lung, cervical, and head and neck squamous cell carcinoma patients as well (Fig. 3H). Altogether, these findings suggest that the increase of DNA-PKcs by CTLA-4 depletion activates the STING pathway to the senescence process in human and mouse melanoma cancer cells, and DNA-PKcs is required for CTLA-4 depletion-induced senescence.

### STING modulates AKT pathway activated by CTLA-4 targeting

**Fig. 4:**
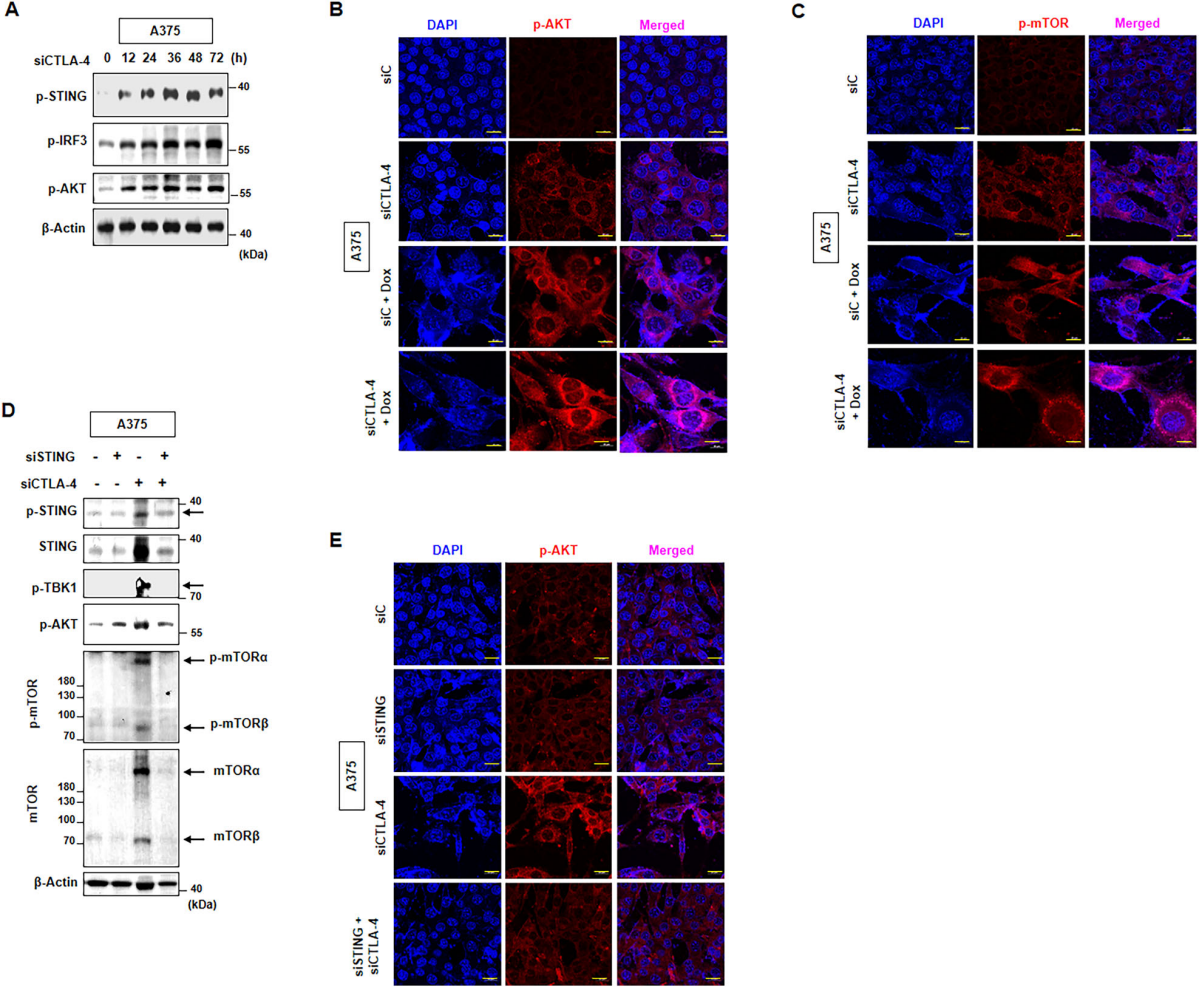
STING modulates AKT pathway activated by CTLA-4 targeting. A375 cells were transfected with CTLA-4 siRNA for indicated times, and WB with indicated antibodies (**A**) confocal microscopy assay for p-AKT (**B**) p-mTOR (**C**) were performed on day two post Dox treatment. siAKT was transfected one day before CTLA-4 siRNA treatment and WB with indicated antibodies (**D**) and confocal microscopy assay (**E**) with indicated antibodies were performed on day three post CTLA-4 siRNA treatment. Scale bars, 20 μm (**B**, **C**, **E**).

STING activation can lead to metabolic adaptations in cells, such as the activation of ACLY (ATP-citrate lyase), which is involved in lipid synthesis and energy production [34]. This metabolic reprogramming can impact the AKT pathway, which is involved in cell cycle [35], senescence [36], and autophagy [37]. So, we investigated the status of the AKT pathway after CTLA-4 siRNA transfection. Interestingly, elevated p-AKT was accompanied by p-STING and p-IRF3 elevation in a time-dependent manner post CTLA-4 siRNA transfection (Fig. 4A). In addition, the expression of p-AKT and the downstream target of AKT, p-mTOR were increased, as assessed by immunofluorescence assay, by CTLA-4 siRNA and were further enhanced by Dox co-treatment (Figs. 3B, C, and S3A–C). p-mTORα and p-mTORβ, which are the full-length and the splicing isoform of mTOR, respectively, and p-mTORβ is considered an active protein kinase beyond the full-length mTOR [38], were elevated by CTLA-4 siRNA. However, the upregulation of p-AKT and p-mTOR by CTLA-4 siRNA treatment were restored to baseline level when siSTING was treated (Figs. 4D, E and S3D). Overall, these findings indicate that STING pathway controls AKT pathway, which is activated by CTLA-4 targeting.

### CTLA-4-depletion impedes tumor growth via senescence induction

**Fig. 5:**
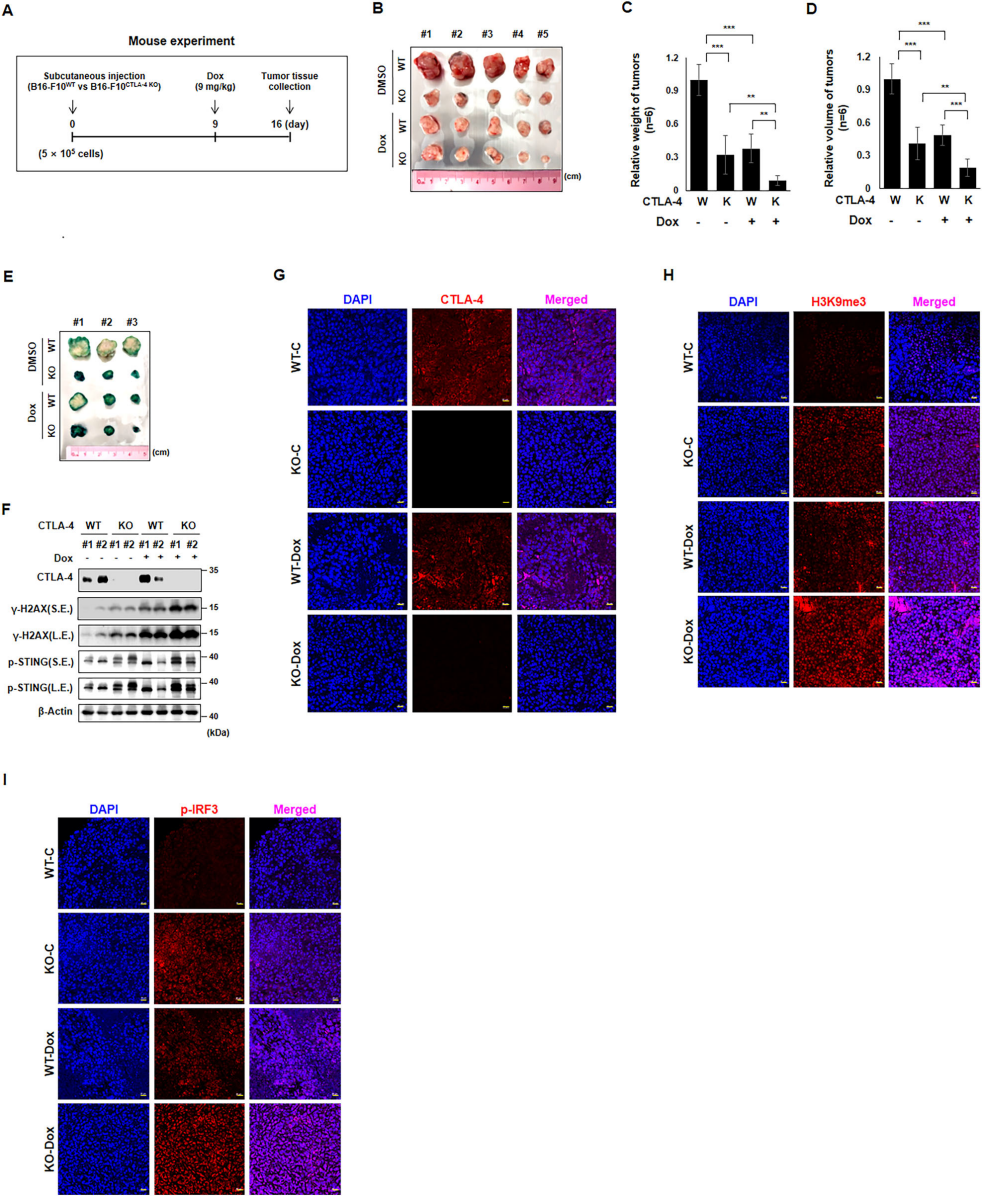
Absence of CTLA-4 impairs tumor growth via senescence induction in vivo. Tumors were generated by implanting B16-F10^wt^ or B16-F10^CTLA-4 KO^ cells (5 × 10^5^) into both dorsal flank regions of mice. On day nine after implantation, mice were administered Dox (9 mg/kg) for seven days, and tumors were excised (**A**). The images of the autopsied tumors (*n* = 5) (**B**). Tumor weights (*n* = 6) (**C**) and volume (*n* = 6) (**D**) were measured. Initially, five sets of autopsied tumor were obtained and photographed (**B**) and subsequently, data from an additional set were included for weight and volume measurements (**C**, **D**). Tumors were cut in half and analyzed by SA-β-Gal staining (*n* = 3) (**E**). WB was performed with CTLA-4 WT vs KO tumors with or without Dox treatment with indicated antibodies for each group (*n* = 2) (**F**). Confocal microscopy assay was performed with tumors for individual CTLA-4, H3K9me3, and p-IRF3 (**G**–**I**). Scale bars, 20 μm (**G**–**I**). The significance of the statistical difference was calculated using a one-way analysis of variance and Newman–Keuls; *p < 0.05. **p < 0.01. ***p < 0.001.

Finally, we performed mouse experiments to validate the implications of previous findings on tumor growth. We hired the B16-F10^CTLA-4 KO^ cell line. C57BL/6 female mice were subcutaneously injected with 5 × 10^5^ cells of B16-F10^WT^ and B16-F10^CTLA-4 KO^ cells into each flank region, followed by intraperitoneal injection of 9 mg/kg Dox at day 9 for the combination effects of CTLA-4 depletion and anti-tumor drug treatment. Tumor masses were collected on day 16 (Fig. 5A). The tumor size, weight, and volume derived from the mice injected with B16-F10^CTLA-4 KO^ cells were significantly lower than those of the tumors derived from the mice injected with B16-F10^WT^ cells (Fig. 5B–D). And the intensity of the SA-β-Gal staining in B16-F10^CTLA-4 KO^-derived tumors was remarkably stronger than that of B16-F10^WT^-derived tumors (Fig. 5E). These results confirm the effect of CTLA-4 deficiency on tumor cell senescence. B16-F10^CTLA-4 KO^-derived tumors showed higher expression of γ-H2AX and p-STING than B16-F10^WT^-derived tumors in both Dox-treated and non-treated groups (Fig. 5F). Immunohistochemistry analysis confirmed that B16-F10^CTLA-4 KO^ tumors showed no CTLA-4 staining (Fig. 5G), and the increased intensities of H3K9me3, a senescence marker, and p-IRF3, a downstream product of cGAS-STING activation, in both Dox non-treated and -treated groups (Fig. 5H, I).

In conclusion, CTLA-4 depletion in cancer cells leads to the formation of micronuclei resulting from nuclear instability. Specifically, this instability, led and exacerbated by Aurora B reduction due to the CTLA-4 depletion, allows cytosolic DNA to activate DNA-PKcs. Subsequently, the DNA-PKcs canonical downstream, STING signaling pathway is activated, which in turn triggers the AKT pathway. This activation then engages the p53-p21 axis, inducing senescence and ultimately leading to tumor growth regression (Fig. 6).

## Discussion

Understanding the role of CTLA-4 in cancer cells, beyond T cells, is crucial for advancing cancer therapy. In the current study, we depleted CTLA-4 expression through silencing and examined its effects in mouse and human melanoma cells. Remarkably, CTLA-4 depletion induced cellular senescence, characterised by irreversible cell cycle arrest, which inhibited cancer cell proliferation. We observed up-regulation of DNA damage and heterochromatin markers, such as γ-H2AX and H3K9me3. We also elucidated the mechanism underlying CTLA-4 depletion-induced senescence. Micronuclei, the broadly known marker of DNA damage and chromosomal instability, are tiny DNA components of the nucleus that are dispersed from the original nucleus and are commonly observed in diseases such as cancer and senescence. In the current study, CTLA-4 depletion in melanoma cells led to genomic instability manifested by the formation of micronuclei. This instability was attributed to the downregulation of Aurora B, a protein critical for maintaining genome stability and preventing micronuclei formation. Furthermore, a recent report has also highlighted a positive correlation between CTLA-4 and Aurora B in patients with hepatocellular carcinoma [39], implying that high Aurora B expression is associated with high CTLA-4 in the tumor microenvironment (TME) and leads to poor prognosis. Subsequently, DNA-PKcs detected this genomic instability and sequentially activated the STING-AKT signaling pathway. DNA-PKcs is critical for maintaining genome integrity [17]. In addition to its canonical function in DNA repair, DNA-PKcs also detects cytosolic DNA and activates the cGAS-STING pathway [19, 20]. Indeed, DNA-PKcs is involved in DNA-driven immune response cGAS-STING in a dependent or independent manner. In our study, the atypical role of DNA-PKcs in senescence induced by CTLA-4 depletion is particularly intriguing. We show that DNA-PKcs activates the STING pathway, as evidenced by the effect of DNA-PKcs silencing in our experiments. This finding is consistent with recent reports showing that specific inhibitors of DNA-PKcs, such as NU7441, suppress the activated STING pathway induced by double-stranded DNA [40]. Overall, the activated STING pathway induced by CTLA-4 deficiency was effectively blocked by inhibition of DNA-PKcs in melanoma cells in our study. In contrast, one of the major DNA damage HR pathways is not related with CTLA-4 in TCGA data from patients (Fig. 3G).

While previous studies have found that CTLA-4 silencing reduces cellular proliferation [41–43] and that CTLA-4 induces apoptosis in cancer cells [44], our study is the first to identify the role of CTLA-4 as a regulator of senescence in cancer. These findings strongly support our hypothesis that the absence of CTLA-4 increases sensitivity to DDR. Collectively, CTLA-4 depletion leads to a decrease in Aurora B levels, resulting in micronuclei formation and DNA damage [45], predisposing cells to enter senescence via activation of the STING pathway. Our study may contribute to a better understanding and potential strategies for CTLA-4–targeted therapies in cancer. Although further studies are needed to elucidate how CTLA-4 deficiency leads to genome instability via Aurora B reduction and how STING activates the AKT pathway, our research has uncovered the role of CTLA-4 in senescence. Although senescence is defined as a permanent cell cycle arrest, which can inhibit tumors, it is also known to contribute to therapeutic resistance by secreting the senescence-associated secretory phenotype (SASP). Inducing senescence and removing SASP are considered favorable strategies to overcome therapy resistance and improve the efficacy of cancer treatment [46, 47]. In this study, we used siCTLA-4 to reduce CTLA-4 expression or CTLA-4 KO cells, as the CTLA-4 inhibitor compound was unavailable. Further investigation using CTLA-4 inhibitor will be necessary for the future therapeutic approach.

Overall, targeting intrinsic CTLA-4 in cancer cells may be a promising therapeutic approach to induce cancer regression through senescence mechanisms. In conclusion, our findings highlight senescence as a prominent response in CTLA-4-depleted melanoma cells, mediated by the DNA-PKcs-STING-AKT-p21 axis.

## Supplementary information

Revised CDDis checklist

Sypplementary information-Clean Version

Original Data Source

## Data Availability

Supplementary Figs. and the source data underlying Figs. are provided as a Supplementary information file. Other figures or data supporting the results of this study are available from the corresponding author upon reasonable request.

## References

[1] Hernandez-Segura A, Nehme J, Demaria M. Hallmarks of cellular senescence. Trends Cell Biol. 2018;28:436–53. 29477613 10.1016/j.tcb.2018.02.001

[2] Sidler C, Kovalchuk O, Kovalchuk I. Epigenetic regulation of cellular senescence and aging. Front Genet. 2017;8:138. 29018479 10.3389/fgene.2017.00138 PMC5622920

[3] Fitsiou E, Soto-Gamez A, Demaria M. Biological functions of therapy-induced senescence in cancer. Semin Cancer Biol. 2022;81:5–13. 33775830 10.1016/j.semcancer.2021.03.021

[4] Oyewole-Said D, Konduri V, Vazquez-Perez J, Weldon SA, Levitt JM, Decker WK. Beyond T-cells: functional characterization of CTLA-4 expression in immune and non-immune cell types. Front Immunol. 2020;11:608024. 33384695 10.3389/fimmu.2020.608024 PMC7770141

[5] Laurent S, Queirolo P, Boero S, Salvi S, Piccioli P, Boccardo S, et al. The engagement of CTLA-4 on primary melanoma cell lines induces antibody-dependent cellular cytotoxicity and TNF-alpha production. J Transl Med. 2013;11:108. 23634660 10.1186/1479-5876-11-108 PMC3663700

[6] Pistillo MP, Carosio R, Grillo F, Fontana V, Mastracci L, Morabito A, et al. Phenotypic characterization of tumor CTLA-4 expression in melanoma tissues and its possible role in clinical response to Ipilimumab. Clin Immunol. 2020;215:108428. 32344017 10.1016/j.clim.2020.108428

[7] Rowshanravan B, Halliday N, Sansom DM. CTLA-4: a moving target in immunotherapy. Blood. 2018;131:58–67. 29118008 10.1182/blood-2017-06-741033 PMC6317697

[8] Loo TM, Miyata K, Tanaka Y, Takahashi A. Cellular senescence and senescence-associated secretory phenotype via the cGAS-STING signaling pathway in cancer. Cancer Sci. 2020;111:304–11. 31799772 10.1111/cas.14266 PMC7004529

[9] Schmitz CRR, Maurmann RM, Guma F, Bauer ME, Barbe-Tuana FM. cGAS-STING pathway as a potential trigger of immunosenescence and inflammaging. Front Immunol. 2023;14:1132653. 36926349 10.3389/fimmu.2023.1132653 PMC10011111

[10] Ou L, Zhang A, Cheng Y, Chen Y. The cGAS-STING pathway: a promising immunotherapy target. Front Immunol. 2021;12:795048. 34956229 10.3389/fimmu.2021.795048 PMC8695770

[11] Dou Z, Ghosh K, Vizioli MG, Zhu J, Sen P, Wangensteen KJ, et al. Cytoplasmic chromatin triggers inflammation in senescence and cancer. Nature. 2017;550:402–6. 28976970 10.1038/nature24050 PMC5850938

[12] Nogueira V, Park Y, Chen CC, Xu PZ, Chen ML, Tonic I, et al. Akt determines replicative senescence and oxidative or oncogenic premature senescence and sensitizes cells to oxidative apoptosis. Cancer Cell. 2008;14:458–70. 19061837 10.1016/j.ccr.2008.11.003 PMC3038665

[13] Lee JJ, Kim BC, Park MJ, Lee YS, Kim YN, Lee BL, et al. PTEN status switches cell fate between premature senescence and apoptosis in glioma exposed to ionizing radiation. Cell Death Differ. 2011;18:666–77. 21072054 10.1038/cdd.2010.139 PMC3131905

[14] Seo GJ, Yang A, Tan B, Kim S, Liang Q, Choi Y, et al. Akt kinase-mediated checkpoint of cGAS DNA sensing pathway. Cell Rep. 2015;13:440–9. 26440888 10.1016/j.celrep.2015.09.007 PMC4607670

[15] Chou WC, Rampanelli E, Li X, Ting JP. Impact of intracellular innate immune receptors on immunometabolism. Cell Mol Immunol. 2022;19:337–51. 34697412 10.1038/s41423-021-00780-y PMC8891342

[16] Qiao J, Zhang Z, Ji S, Liu T, Zhang X, Huang Y, et al. A distinct role of STING in regulating glucose homeostasis through insulin sensitivity and insulin secretion. Proc Natl Acad Sci USA. 2022;119. 10.1073/pnas.2101848119 PMC8851542 35145023

[17] Yue X, Bai C, Xie D, Ma T, Zhou PK. DNA-PKcs: A Multi-Faceted Player in DNA Damage Response. Front Genet. 2020;11:607428. 33424929 10.3389/fgene.2020.607428 PMC7786053

[18] Goodwin JF, Knudsen KE. Beyond DNA repair: DNA-PK function in cancer. Cancer Discov. 2014;4:1126–39. 25168287 10.1158/2159-8290.CD-14-0358 PMC4184981

[19] Hristova DB, Oliveira M, Wagner E, Melcher A, Harrington KJ, Belot A, et al. DNA-PKcs is required for cGAS/STING-dependent viral DNA sensing in human cells. iScience. 2024;27:108760. 38269102 10.1016/j.isci.2023.108760 PMC10805666

[20] Taffoni C, Steer A, Marines J, Chamma H, Vila IK, Laguette N. Nucleic Acid Immunity and DNA Damage Response: New Friends and Old Foes. Front Immunol. 2021;12:660560. 33981307 10.3389/fimmu.2021.660560 PMC8109176

[21] Lee JJ, Kim SY, Kim SH, Choi S, Lee B, Shin JS. STING mediates nuclear PD-L1 targeting-induced senescence in cancer cells. Cell Death Dis. 2022;13:791. 36109513 10.1038/s41419-022-05217-6 PMC9477807

[22] Dimri GP, Lee X, Basile G, Acosta M, Scott G, Roskelley C, et al. A biomarker that identifies senescent human cells in culture and in aging skin in vivo. Proc Natl Acad Sci USA. 1995;92:9363–7. 7568133 10.1073/pnas.92.20.9363 PMC40985

[23] Zhang XH, Rao M, Loprieato JA, Hong JA, Zhao M, Chen GZ, et al. Aurora A, Aurora B and survivin are novel targets of transcriptional regulation by histone deacetylase inhibitors in non-small cell lung cancer. Cancer Biol Ther. 2008;7:1388–97. 18708766 10.4161/cbt.7.9.6415

[24] Lee JJ, Park IH, Rhee WJ, Kim HS, Shin JS. HMGB1 modulates the balance between senescence and apoptosis in response to genotoxic stress. FASEB J. 2019;33:10942–53. 31284735 10.1096/fj.201900288R

[25] Lee JJ, Park IH, Kwak MS, Rhee WJ, Kim SH, Shin JS. HMGB1 orchestrates STING-mediated senescence via TRIM30alpha modulation in cancer cells. Cell Death Discov. 2021;7:28. 33558529 10.1038/s41420-021-00409-z PMC7870821

[26] Mo X, Zhang H, Preston S, Martin K, Zhou B, Vadalia N, et al. Interferon-gamma Signaling in Melanocytes and Melanoma Cells Regulates Expression of CTLA-4. Cancer Res. 2018;78:436–50. 29150430 10.1158/0008-5472.CAN-17-1615 PMC5771950

[27] Vasudevan S, Flashner-Abramson E, Alkhatib H, Roy Chowdhury S, Adejumobi IA, Vilenski D, et al. Overcoming resistance to BRAF(V600E) inhibition in melanoma by deciphering and targeting personalized protein network alterations. NPJ Precis Oncol. 2021;5:50. 34112933 10.1038/s41698-021-00190-3 PMC8192524

[28] Bigenwald C, Le Berichel J, Wilk CM, Chakraborty R, Chen ST, Tabachnikova A, et al. BRAF(V600E)-induced senescence drives Langerhans cell histiocytosis pathophysiology. Nat Med. 2021;27:851–61. 33958797 10.1038/s41591-021-01304-x PMC9295868

[29] Tran SL, Rizos H. Monitoring oncogenic B-RAF-induced senescence in melanocytes. Methods Mol Biol. 2013;965:313–26. 23296668 10.1007/978-1-62703-239-1_21

[30] Hirota T, Lipp JJ, Toh BH, Peters JM. Histone H3 serine 10 phosphorylation by Aurora B causes HP1 dissociation from heterochromatin. Nature. 2005;438:1176–80. 16222244 10.1038/nature04254

[31] Marima R, Hull R, Penny C, Dlamini Z. Mitotic syndicates Aurora Kinase B (AURKB) and mitotic arrest deficient 2 like 2 (MAD2L2) in cohorts of DNA damage response (DDR) and tumorigenesis. Mutat Res Rev Mutat Res. 2021;787:108376. 34083040 10.1016/j.mrrev.2021.108376

[32] Li Y, Xu FL, Lu J, Saunders WS, Prochownik EV. Widespread genomic instability mediated by a pathway involving glycoprotein Ib alpha and Aurora B kinase. J Biol Chem. 2010;285:13183–92. 20157117 10.1074/jbc.M109.084913 PMC2857109

[33] Burleigh K, Maltbaek JH, Cambier S, Green R, Gale M, Jr., et al. Human DNA-PK activates a STING-independent DNA sensing pathway. Sci Immunol. 2020;5:eaba4219. 10.1126/sciimmunol.aba4219 PMC7081723 31980485

[34] Nickenig M, Mangan MSJ, Lee HE, Symeonidis K, Henne A, Kaiser R, et al. Cutting edge: STING induces ACLY activation and metabolic adaptations in human macrophages through TBK1. J Immunol. 2024;212:7–11. 38038390 10.4049/jimmunol.2200835

[35] Liu P, Begley M, Michowski W, Inuzuka H, Ginzberg M, Gao D, et al. Cell-cycle-regulated activation of Akt kinase by phosphorylation at its carboxyl terminus. Nature. 2014;508:541–5. 24670654 10.1038/nature13079 PMC4076493

[36] Minamino T, Miyauchi H, Tateno K, Kunieda T, Komuro I. Akt-induced cellular senescence: implication for human disease. Cell Cycle. 2004;3:449–51. 15004530

[37] Heras-Sandoval D, Perez-Rojas JM, Hernandez-Damian J, Pedraza-Chaverri J. The role of PI3K/AKT/mTOR pathway in the modulation of autophagy and the clearance of protein aggregates in neurodegeneration. Cell Signal. 2014;26:2694–701. 25173700 10.1016/j.cellsig.2014.08.019

[38] Panasyuk G, Nemazanyy I, Zhyvoloup A, Filonenko V, Davies D, Robson M, et al. mTORbeta splicing isoform promotes cell proliferation and tumorigenesis. J Biol Chem. 2009;284:30807–14. 19726679 10.1074/jbc.M109.056085 PMC2781479

[39] Zhao H, Wang Y, Yang Z, Wei W, Cong Z, Xie Y. High expression of aurora kinase B predicts poor prognosis in hepatocellular carcinoma after curative surgery and its effects on the tumor microenvironment. Ann Transl Med. 2022;10:1168. 36467342 10.21037/atm-22-4798 PMC9708486

[40] Taffoni C, Marines J, Chamma H, Guha S, Saccas M, Bouzid A, et al. DNA damage repair kinase DNA-PK and cGAS synergize to induce cancer-related inflammation in glioblastoma. EMBO J. 2023;42:e111961. 36574362 10.15252/embj.2022111961 PMC10068334

[41] Huang PY, Guo SS, Zhang Y, Lu JB, Chen QY, Tang LQ, et al. Tumor CTLA-4 overexpression predicts poor survival in patients with nasopharyngeal carcinoma. Oncotarget. 2016;7:13060–8. 26918337 10.18632/oncotarget.7421 PMC4914341

[42] Ciszak L, Frydecka I, Wolowiec D, Szteblich A, Kosmaczewska A. CTLA-4 affects expression of key cell cycle regulators of G0/G1 phase in neoplastic lymphocytes from patients with chronic lymphocytic leukaemia. Clin Exp Med. 2016;16:317–32. 26003188 10.1007/s10238-015-0360-7 PMC4969362

[43] Greenwald RJ, Oosterwegel MA, van der Woude D, Kubal A, Mandelbrot DA, Boussiotis VA, et al. CTLA-4 regulates cell cycle progression during a primary immune response. Eur J Immunol. 2002;32:366–73. 11807776 10.1002/1521-4141(200202)32:2<366::AID-IMMU366>3.0.CO;2-5

[44] Yan Q, Zhang B, Ling X, Zhu B, Mei S, Yang H, et al. CTLA-4 facilitates DNA damage-induced apoptosis by interacting with PP2A. Front Cell Dev Biol. 2022;10:728771. 35281086 10.3389/fcell.2022.728771 PMC8907142

[45] Kwon M, Leibowitz ML, Lee JH. Small but mighty: the causes and consequences of micronucleus rupture. Exp Mol Med. 2020;52:1777–86. 33230251 10.1038/s12276-020-00529-z PMC8080619

[46] Xiao S, Qin D, Hou X, Tian L, Yu Y, Zhang R, et al. Cellular senescence: a double-edged sword in cancer therapy. Front Oncol. 2023;13:1189015. 37771436 10.3389/fonc.2023.1189015 PMC10522834

[47] Feng T, Xie F, Lee LMY, Lin Z, Tu Y, Lyu Y, et al. Cellular senescence in cancer: from mechanism paradoxes to precision therapeutics. Mol Cancer. 2025;24:213. 40781676 10.1186/s12943-025-02419-2 PMC12333312

