# Supplementary material for: DNA-PKcs orchestrates CTLA-4 depletion-induced senescence in cancer cells
Source: Cell Death Dis. 2026 Feb 4;17(1):204. doi: 10.1038/s41419-026-08419-4 (PMC12895018; doi:10.1038/s41419-026-08419-4)

**Supplementary Information**

**DNA-PKcs orchestrates CTLA-4 depletion-induced senescence**

**in cancer cells**

**Je-Jung Lee ^#1, 2, *^, Woo Joong Rhee ^#1, 2^**, **So Young Kim ^1, 3^, Jisun Lee ^1, 3^, Su Ful Jung ^1, 3^, Jooyeon Oh ^1, 3^, In Ho Park ^2, 4^, Jeon-Soo Shin ^1–3, *^**

^1^Department of Microbiology, Yonsei University College of Medicine, Seoul, South Korea.

^2^Institute for Immunology and Immunological Diseases, Yonsei University College of Medicine, Seoul, South Korea.

^3^Brain Korea 21 FOUR Project for Medical Science, Yonsei University College of Medicine, Seoul, South Korea.

^4^Center for Nanomedicine, Institute for Basic Science (IBS), Yonsei University, Seoul, South Korea.

**^#^Contributed equally.**

^*^**Corresponding Authors**:

Jeon-Soo Shin: Department of Microbiology, Yonsei University College of Medicine, 50-1 Yonsei-ro, Seodaemun-gu, Seoul 03722, South Korea

Tel: +82-2-2228-1816, Fax: +82-2-392-7088, E-mail: [jsshin6203@yuhs.ac](mailto:jsshin6203@yuhs.ac)

Je-Jung Lee: Department of Microbiology, Yonsei University College of Medicine, 50-1 Yonsei-ro, Seodaemun-gu, Seoul 03722, South Korea

Tel: +82-2-2228-0800, Fax: +82-2-392-7088

**Supplementary Figures**

**
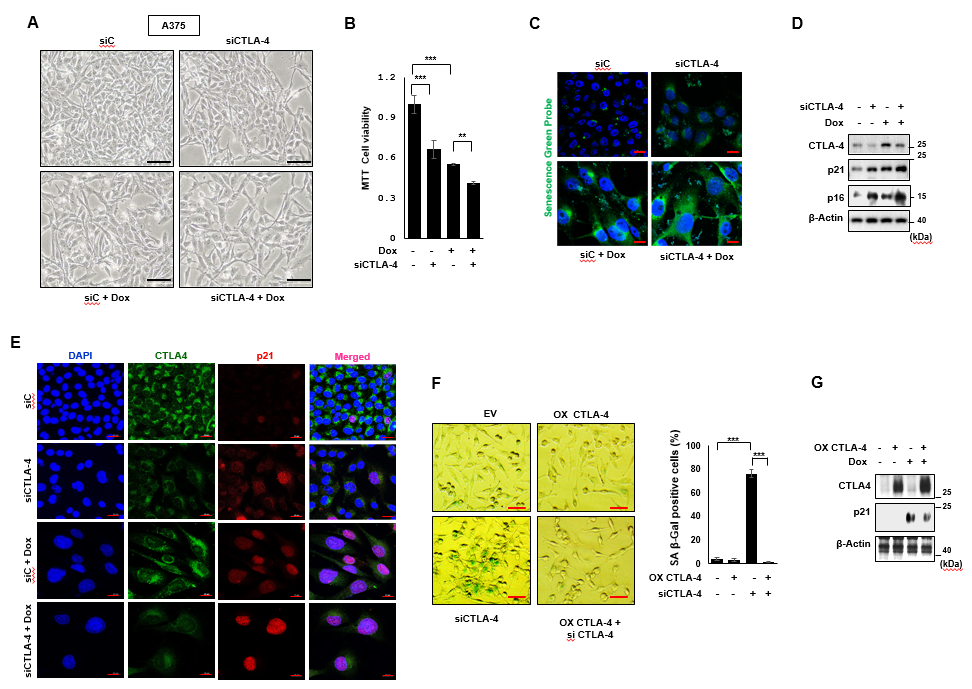

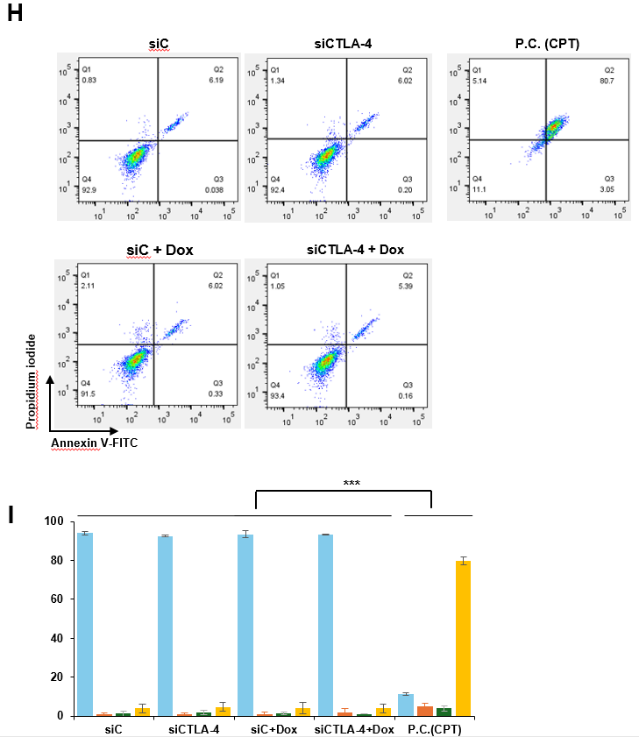

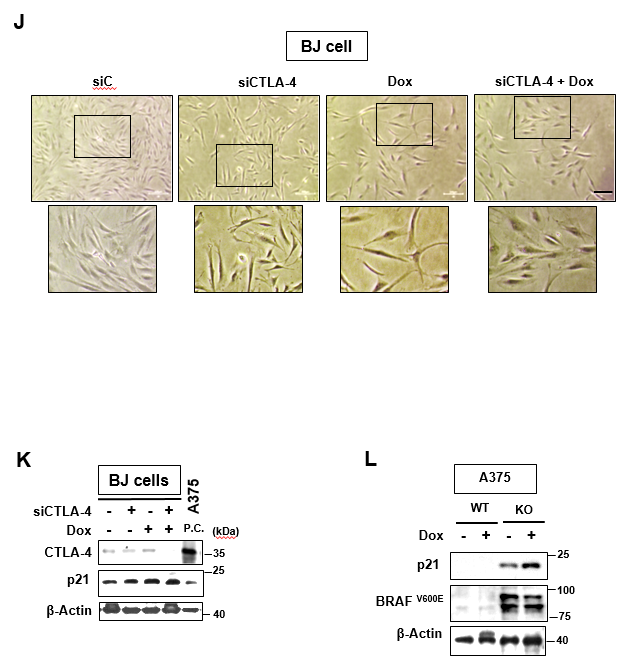
**

**Fig S1. CTLA-4 depletion induces Senescence in cancer cells.** A375 cells were transfected with 100 nM siC and CTLA-4 siRNA, respectively, one day before treatment with or without 100 ng/ml of Dox. Subsequently, morphological changes were detected (A), Analysis of cell viability by MTT assay (B), SA-β-Gal staining detected by Senescent Green Probe (C), WB analysis with indicated antibodies (D), Confocal microscopy assay for examination of p21, CTLA-4, and DAPI levels performed on day two after Dox treatment (E). CTLA-4 was overexpressed one day before treatment with or without Dox treatment. Then SA-β-Gal staining with quantification (F), WB with indicated antibodies (G) were performed on day two post Dox treatment. Annexin V/PI staining assay was performed by FACS analysis after CTLA-4 siRNA and Dox treatment, either separately or together (H), and the results were quantified (I). Human skin BJ cells were treated with CTLA-4 siRNA for one day, either with or without Dox treatment. The morphological changes were observed on day two after Dox treatment (J), as was the WB assay (K). BRAF^V600E^ levels are elevated in CTLA-4 depletion-induced senescence. CTLA-4 WT and KO A375 cells were treated with Dox for two days, and WB analysis was performed using the indicated antibodies (L).

Scale bars, 50 μm (A, F), 20 μm (C, E), and 200 μm (J). The significance of the statistical differences among the four groups was calculated using a one-way analysis of variance and Newman-Keuls methods. Quantitative data are expressed as means ± SD. N = 3, **p* < 0.05. ***p* < 0.01. ****p* < 0.001.


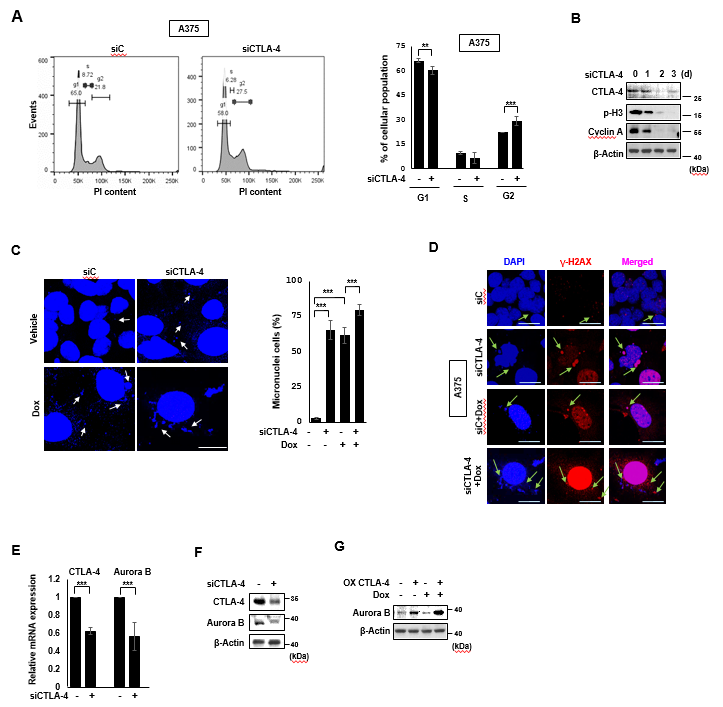


**Fig S2. AURKB reduction by CTLA-4 depletion leads cell cycle arrest via genome instability in cancer cells.** A375 cells were transfected with CTLA-4 siRNA, then cell cycle analysis with quantification on day 3 by FACS (A), WB as indicated times with indicated antibodies from day 0 to 3 (B) was performed. CTLA-4 siRNA was transfected one day before Dox treatment and confocal microscopy assay for micronuclei by DAPI staining with quantification (C), for γ-H2AX (D) were performed on day two post Dox treatment. Cells were treated with CTLA-4 siRNA and real time PCR assay (E) and WB (F) for CTLA-4 and Aurora B were performed on day two. CTLA-4 was overexpressed one day before Dox treatment and WB was performed with indicated antibodies on day two (G). Scale bars, 20 μm (C, D). The significance of the statistical differences among the four groups was calculated using a one-way analysis of variance and Newman-Keuls methods. Quantitative data are expressed as means ± SD. N = 3, **p* < 0.05. ***p* < 0.01. ****p* < 0.001.

**
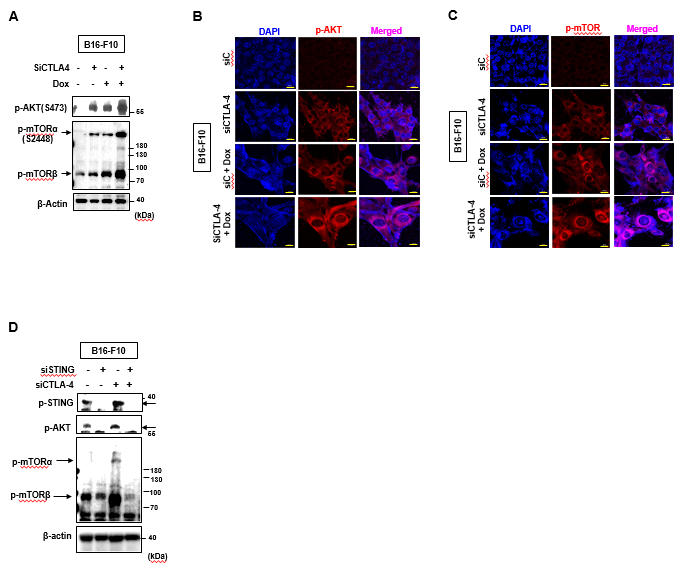
**

**Fig S3. STING modulates AKT pathway activated by CTLA-4 targeting.** B16-F10 cells were transfected with CTLA-4 siRNA one day before Dox treatment and WB with indicated antibodies (A), confocal microscope assay for p-AKT (B), p-mTOR (C) were performed on day two post Dox treatment. siSTING was treated one day before CTLA-4 siRNA transfection and WB with indicated antibodies was performed at indicated times on day two (D). Scale bars, 20 μm (B, C).

**Supplementary Table**

**Materials Lists**

1. Penicillin-streptomycin (Life Technologies, Waltham, MA, USA)
2. CLLA-4 plasmid (HG11159-NY) and Aurora B (LPO-MG53910-CM) (Sino Biological, Wayne, PA, USA) were obtained from Sino Biological (Wayne, PA, USA).
3. FuGene HD reagent (Promega, Madison, WI, USA)
4. RNAiMAX (Invitrogen, Carlsbad, CA, USA)
5. siRNA duplexes against mouse CTLA-4 (sc-42767), DNA-PK (sc-35201) (Santa Cruz Biotechnology, Dallas, TX, USA)
6. Dox (Calbiochem, San Diego, CA, USA)
7. Cisplatin (P4394) (Sigma-Aldrich, Burlington, MA, USA)
8. Nu 7441 (S2638), KU-60019 (S1570) (Selleck Chemicals, Houston, TX, USA)
9. Horseradish peroxidase (HRP)-conjugated anti-rabbit and anti-mouse antibodies (Santa Cruz Biotechnology, Dallas, TX, USA)
10. Antibodies against p-IRF3 (29047), STING (13647), p-TBK1 (5483), antibodies (Cell Signaling Technologies, Inc. Danvers, MA, USA)
11. p-AKT (4058), AKT (9272), p-mTOR (2971), mTOR (2983), β-actin (4967), tri-H3K9 (13969), p53 (S15) (9284), secondary antibodies (Cell Signaling Technologies, Inc., Danvers, MA, USA)
12. DNA-PKcs (ab32566), γ-H2AX (ab11174), p16 (ab51243) (Abcam, Cambridge, MA, USA),
13. p21(556431), p27(610242) (BD Biosciences, Franklin Lakes, NJ, USA)
14. CTLA-4 (ab237712) (Abcam, Cambridge, MA, USA).
15. p53 (sc-47698) (Santa Cruz Biotechnology, Dallas, TX, USA)
16. p-STING (AF7416) (Affnit, Nottingham, England)
17. Normal rabbit IgG or normal mouse IgG (Santa Cruz Biotechnology, USA)
18. Protein A magnetic beads (Millipore, Billerica, MA, USA)
19. CELLEventTM Senescence Green Detection Kit, C10850, (Invitrogen, Waltham, MA, USA
20. GraphPad Prism 5 (GraphPad Software, Inc., La Jolla, CA, USA)
21. Bioneer, Inc. (Daejeon, Korea).
22. BioLegend (Seoul, Korea)
23. Calbiochem (San Diego, CA, USA).
24. Selleck Chemicals (Houston, TX, USA).
25. an inverted phase-contrast microscope (Olympus, Tokyo, Japan)
26. BD FACS verse II (Becton, Dickinson and Company, Franklin Lakes Enterprises, NJ, USA).
27. FLOWJO, Single Cell Analysis Software v10 (OR, USA).
28. BRAFV600E (SAB5600047) antibody (MilliporeSigma/Sigma-Aldrich, MO, USA)
29. Anti-human CTLA-4 (Ipilimumab Biosimilar) antibody (Bio X Cell, NH, USA)

**Abbreviation**

1. Cytotoxic T-lymphocyte–associated protein 4 (CTLA-4)
2. Cyclic GMP–AMP synthase (cGAS)
3. Stimulator of interferon genes (STING)
4. TANK-binding kinase 1 (TBK1)
5. Interferon regulatory factor 3 (IRF3)
6. Cytoplasmic chromatin fragments (CCFs)
7. DNA-dependent protein kinase catalytic subunit (DNA-PKcs)
8. DNA double strand breaks (DSB)
9. Non-homologous end joining (NHEJ)
10. DNA damage repair (DDR)
11. Dulbecco’s Modified Eagle’s medium (DMEM)
12. The Cancer Genome Atlas (TCGA)
13. Oncogene-induced senescence (OIS)
14. Wild type (WT)
15. Knockout (KO)
16. Overexpression (OX)
17. H3K9 trimethylation (H3K9me3)
18. Doxorubicin (Dox)
19. Chromatin immunoprecipitation (ChIP)
20. The Cancer Genome Atlas (TCGA)
21. Phospho-histone 3 (p-H3)
22. Senescence-Associated Secretory Phenotype (SASP).

**siRNA sequence**

siControl

Sense: CCUACGCCACCAAUUUCGU

AntiSense: ACGAAAUUGGUGGCGUAGG

CTLA-4 siRNA(h)

Sense: CCCAAAUUACGUGUACUAC

AntiSense: GUAGUACACGUAAUUUGGG

siDNA-PKcs (h)

Sense: GAUCGCACCUUACUCUGUU

AntiSense: AACAGAGUAAGGUGCGAUC

siAKT (m)

Sense: UGCCCUUCUACAACCAGGA

AntiSense: UCCUGGUUGUAGAAGGGCA

siAKT (h)

Sense: GCACUUUCGGCAAGGUGAU

AntiSense: AUCACCUUGCCGAAAGUGC

siSTING (m)

Sense: GGAUCCGAAUGUUCAAUCA

AntiSense: UGAUUGAACAUUCGGAUCC

siSTING (h)

Sense: CCUCAUCAGUGGAAUGGAA

AntiSense: UUCCAUUCCACUGAUGAGG

**Primer sequence for Real time PCR**

CTLA-4 (m)-F: GTACCTCTGCAAGGTGGAACTC

CTLA-4 (m)-R: CCAAAGGAGGAAGTCAGAATCCG

AurB (m)-F: CTTCTACGACCAGCAGAGGATC

AurB (m)-R: GGCATCTGACAGTTCCTCCATG

β-actin (m)-F: CGCCACCAGTTCGCCATGGA

βactin (m)-R: TACAGCCCGGGGAGCATCGT

CTLA-4 (h)-F: ACGGGACTCTACATCTGCAAGG

CTLA-4 (h)-R: GGAGGAAGTCAGAATCTGGGCA

AurB (h)-F: GATGACTTTGAGATTGGGCG

AurB (h)-R: GGGACTTGAAGAGGACCTTG

GAPDH (h)-F: GCACCGTCAAGGCTGAGAAC

GAPDH (h)-R: TGGTGAAGACGCCAGTGGA

**Primer sequence for ChIP assay**

**AurB (h) gene promoter**

F: GGGGAATTTGGGGAAACTT

R: CCAAGGCACTGCTACTCTCC

**Data Source**


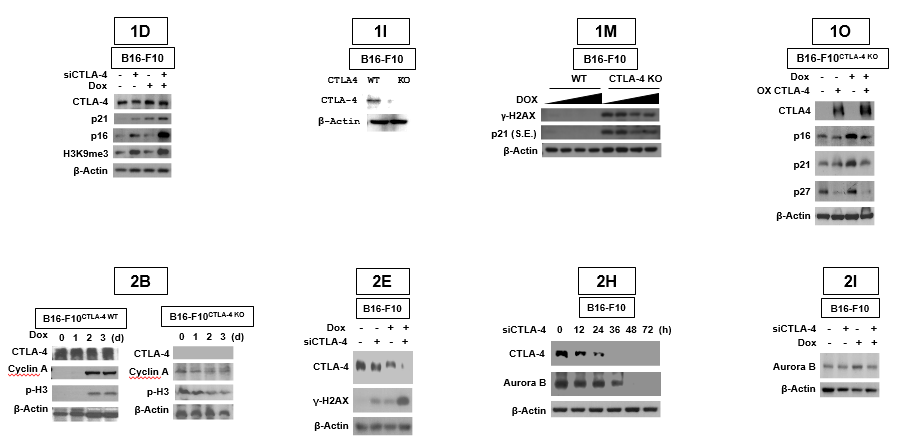


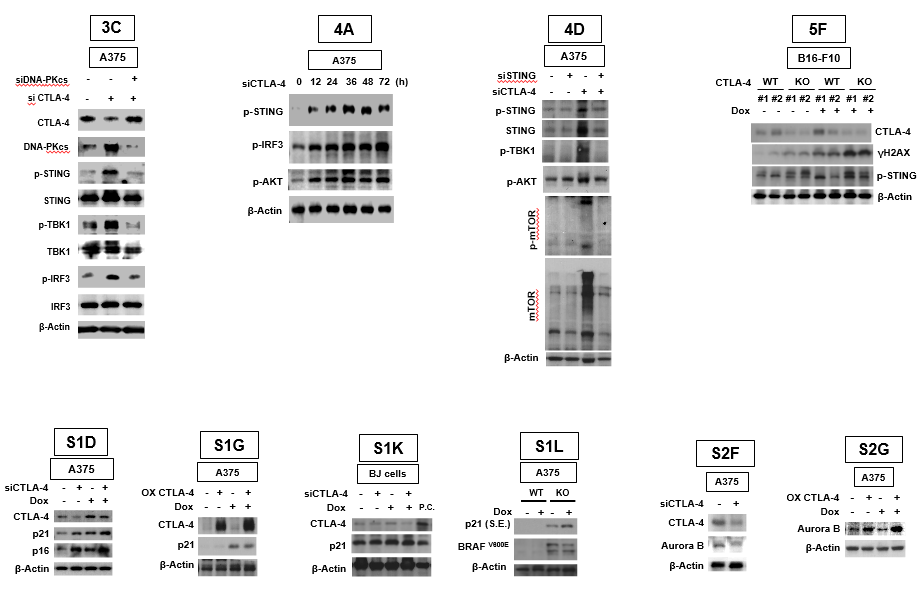


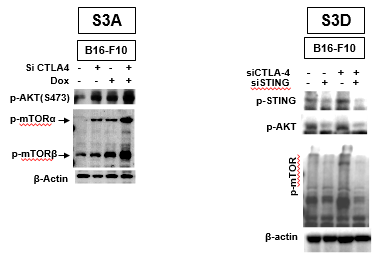

Supplement: Supplementary file 2 — Sypplementary information-Clean Version [file 41419_2026_8419_MOESM2_ESM.docx]
