# Supplementary figures and images for: DNA-PKcs orchestrates CTLA-4 depletion-induced senescence in cancer cells
Source: Cell Death Dis. 2026 Feb 4;17(1):204. doi: 10.1038/s41419-026-08419-4 (PMC12895018; doi:10.1038/s41419-026-08419-4)

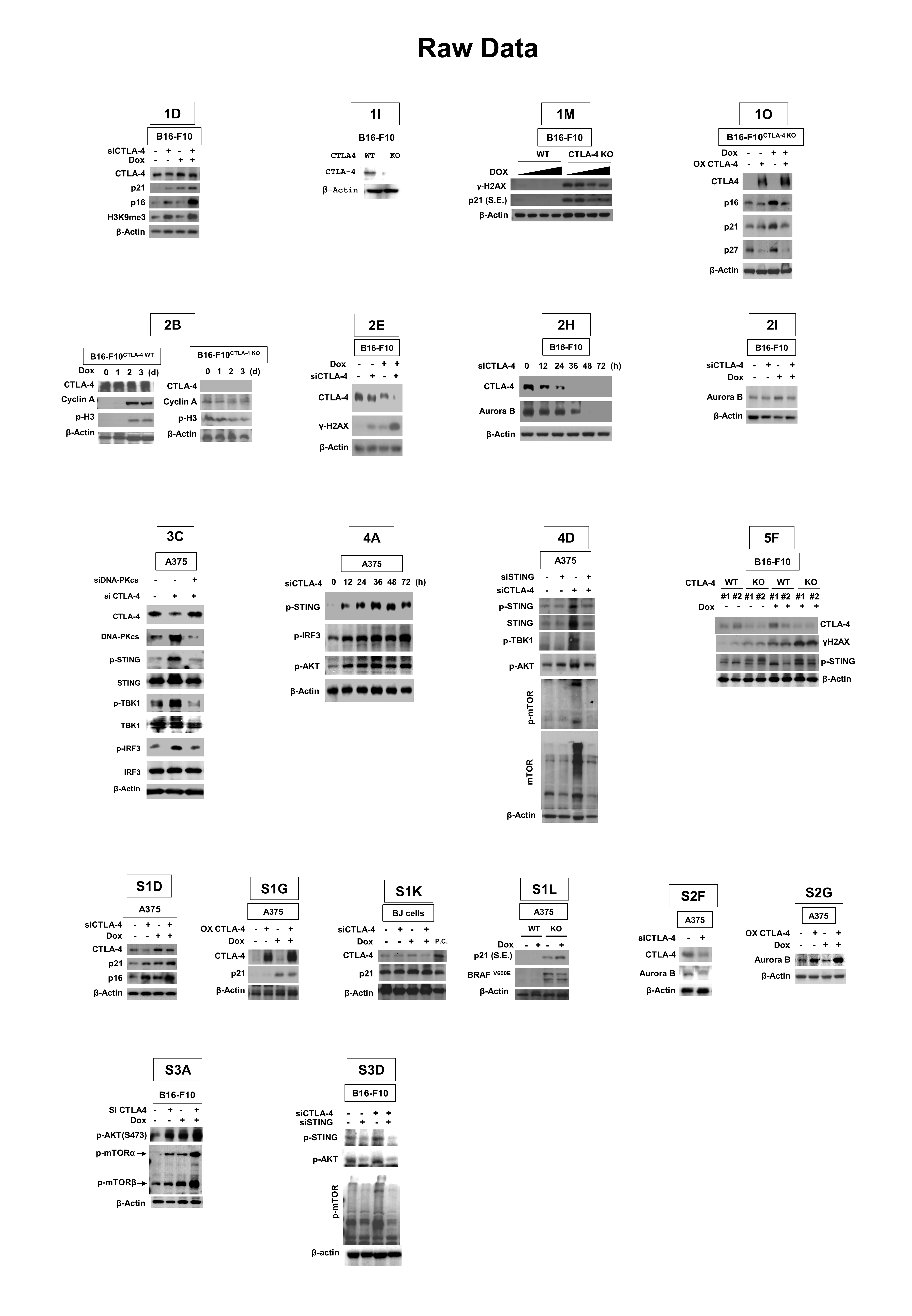

Supplement: Supplementary file 3 — Original Data Source [file 41419_2026_8419_MOESM3_ESM.tif]
